# Diagnostic Accuracy Rate of Tertiary Center Colposcopy Unit

**DOI:** 10.1155/ancp/9307280

**Published:** 2026-06-11

**Authors:** Emre Sertel

**Affiliations:** ^1^ Department of Obstetrics and Gynecology, Kocaeli City Hospital, Kocaeli, Türkiye

**Keywords:** cervical biopsy, cervical cancer, colposcopy, LEEP

## Abstract

**Introduction:**

Colposcopy plays a central role in the evaluation of cervical intraepithelial lesions; however, its diagnostic performance varies widely. While high sensitivity is desirable, low specificity may lead to unnecessary excisional procedures. This study aimed to evaluate the diagnostic performance of colposcopy and its potential contribution to overtreatment in a tertiary referral center.

**Methods:**

This retrospective single‐center study included 150 patients who underwent LEEP between April 2023 and September 2024. Colposcopic impressions were compared with LEEP histopathological outcomes. Sensitivity, specificity, accuracy, positive predictive value (PPV), negative predictive value (NPV), and likelihood ratios were calculated. A subgroup analysis was performed for HPV16‐positive patients.

**Results:**

Colposcopy demonstrated high sensitivity (93.9%, 95% CI: 87.3–97.4) but low specificity (31.4%, 95% CI: 20.2–45.0), with an overall accuracy of 72.7% (95% CI: 65.0–79.2). The PPV and NPV were 72.6% (95% CI: 64.2–79.7) and 72.7% (95% CI: 51.9–86.9), respectively. The positive and negative likelihood ratios were 1.37 and 0.19. Notably, 35 patients (23.3%) were false positives.

In HPV16‐positive patients, sensitivity was 80.8% (95% CI: 71.7–87.5), specificity was 33.3% (95% CI: 21.9–47.1), and accuracy was 64.7% (95% CI: 56.7–72.0).

**Conclusion:**

Although colposcopy is highly sensitive, its low specificity may result in substantial overtreatment, particularly in tertiary referral settings.

## 1. Introduction

Cervical cancer remains one of the most common gynecological malignancies worldwide, representing a significant cause of morbidity and mortality in women [1]. Effective screening programs and accurate diagnostic strategies are essential to reduce disease burden and improve clinical outcomes [2, 3].

Colposcopy is widely used to evaluate cervical lesions and guide targeted biopsy, whereas Loop Electrosurgical Excision Procedure (LEEP) is performed for both diagnostic confirmation and therapeutic excision of abnormal cells [4]. The concordance between colposcopic findings and LEEP histopathology is critical for appropriate clinical management and risk stratification [5].

According to the 2019 ASCCP guidelines, cervical intraepithelial neoplasia grade 1 (CIN1) lesions generally regress spontaneously, whereas CIN2 and CIN3 lesions possess higher malignant potential and warrant treatment [3, 6–8]. However, overtreatment remains a clinical concern, as excisional procedures such as LEEP are associated with potential complications, including intraoperative bleeding, infection, cervical stenosis, and adverse obstetric outcomes [4, 9–11].

This study aimed to evaluate the diagnostic accuracy of colposcopy and its potential role in overtreatment. Despite its widespread adoption, the diagnostic performance of colposcopy remains variable, with sensitivity and specificity influenced by examiner experience, patient population, and lesion characteristics [5, 12]. Tertiary referral centers, in particular, may exhibit distinct diagnostic patterns due to higher disease prevalence and referral bias, underscoring the importance of evaluating real‐world colposcopy performance to optimize clinical decision‐making [6, 9].

## 2. Materials and Methods

### 2.1. Study Design and Setting

This retrospective, single‐center study was conducted at the Department of Obstetrics and Gynecology, Kocaeli City Hospital, Kocaeli, Türkiye, between April 2023 and September 2024. Ethical approval was obtained from the Kocaeli City Hospital Scientific Research Ethics Committee (approval number 2024–108). The study followed the principles of the Declaration of Helsinki, and patient confidentiality was maintained through anonymization of electronic medical records [1, 2].

### 2.2. Patient Selection

Inclusion criteria were:

Age ≥24 years [3].
Non‐pregnant status at colposcopy [4].
Indication for LEEP due to abnormal cervical cytology, positive high‐risk HPV, or suspicious colposcopic findings [5, 6].

Exclusion criteria:

Pregnancy.
History of cervical surgery (conization or hysterectomy).
Inadequate colposcopic visualization.
A total of 150 consecutive patients met these criteria and were included.

### 2.3. Colposcopy Procedure

Colposcopies were performed by gynecologic oncology specialists, following the 2019 ASCCP Colposcopy Standards [5]. All examinations were conducted by experienced colposcopists with at least 10 years of expertise in cervical pathology and colposcopic evaluation. All examinations were documented using standard colposcopic terminology [9], recording impressions as:

HSIL: High‐grade lesion suspected.
Other: Normal or low‐grade lesion.

Adequacy was assessed based on visibility of the transformation zone and quality of the examination, consistent with international standards [5, 9, 12].

### 2.4. Cytology and HPV Testing

Cervical cytology was classified using the Bethesda system [13]:

NILM (negative for intraepithelial lesion or malignancy).
ASC‐US (atypical squamous cells of undetermined significance).
LSIL (low‐grade squamous intraepithelial lesion).
HSIL (high‐grade squamous intraepithelial lesion).
ASC‐H (cannot exclude HSIL).
AGC (atypical glandular cells).

HPV testing was performed using PCR–based assays for high‐risk types, including HPV16, HPV18, and other high‐risk HPV types [6, 14–16].

### 2.5. Loop Electrosurgical Excision Procedure (LEEP)

LEEP was performed for diagnostic and/or therapeutic purposes under local or general anesthesia, following standard protocols [4, 17]. Specimens were properly oriented, labeled, and submitted for histopathological evaluation. Lesions were classified as:

LSIL: Low‐grade squamous intraepithelial lesion.
HSIL: High‐grade squamous intraepithelial lesion.

In patients with ASC‐US cytology, LEEP was performed in selected cases based on additional risk factors, including persistent cytological abnormalities, high‐risk HPV positivity (particularly HPV16), suspicious colposcopic findings, or clinical decision‐making in a tertiary referral setting. This reflects real‐world practice, where management is guided by cumulative risk assessment rather than cytology alone.

### 2.6. Data Collection

Demographic, clinical, and pathological data were extracted from the Hospital Management System (KEYDATA), including:

Age, gravida, parity.
Pap smear results.
HPV status.
Colposcopy impressions.
LEEP histopathology.

LEEP histopathology was accepted as the reference standard (gold standard) for evaluating the diagnostic performance of colposcopy.

### 2.7. Statistical Analysis

Sensitivity, specificity, accuracy, PPV, and NPV were calculated for the whole cohort and HPV16‐positive subgroup [18, 19]. Sensitivity was calculated as TP/(TP + FN), and specificity as TN/(TN + FP). Positive predictive value (PPV) was defined as TP/(TP + FP), and negative predictive value (NPV) as TN/(TN + FN).

95% confidence intervals were calculated using the binomial exact method [18].

Likelihood ratios (LR+ and LR−) were estimated [19].

A positive colposcopy result was defined as an impression suggestive of HSIL. Likelihood ratios were calculated using standard formulas:

LR+=Sensitivity/1−Specificity,

and

LR−=1−Sensitivity/Specificity.

Correlations between cytology and LEEP histopathology were evaluated descriptively.

Analyses were performed using SPSS version 25.0 (IBM Corp., Armonk, NY, USA).

### 2.8. Subgroup Analysis

Subgroup analyses were performed for:

HPV16‐positive patients [6].
Cytology subgroups (ASC‐US, LSIL, HSIL).
Age strata (<40 vs ≥40).

This allowed evaluation of colposcopy performance in clinically relevant subsets [5, 9, 17].

### 2.9. Ethical Considerations

Written informed consent was waived due to the retrospective design. All procedures adhered to ethical standards, and patient confidentiality was strictly maintained [1, 2].

## 3. Results

### 3.1. Patient Characteristics

**Figure 1:**
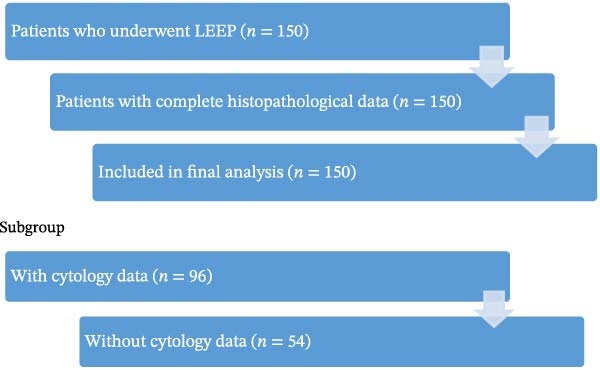
Flow diagram of patient inclusion and data availability in the study.

A total of 150 patients were included (Figure 1). The mean age was 41.9 ± 8.3 years (range 24–72), with a mean gravida of 2.3 ± 1.3 and mean parity of 1.8 ± 1.1. Regarding HPV status, 114 patients (76.0%) were HPV16 positive, 19 patients (12.7%) were HPV18 positive, and 38 patients (25.3%) had other high‐risk HPV types. Cytology distribution included 30 patients (20%) with normal cytology, 28 patients (18.6%) with ASC‐US, 18 patients (12%) with LSIL, 8 patients (5.3%) with HSIL, 10 patients (6.6%) with ASC‐H, 2 patients (1.3%) with AGC, and 54 patients (36%) had no available smear results. (Refer to Table 1 for detailed demographic and cytology data.)

**Table 1:** Demographic and clinical characteristics, HPV and Pap smear results.

Characteristic	*n*	%/Mean ± SD/Range
Age	—	41.9 ± 8.3 (24–72)
Gravida	—	2.3 ± 1.3 (0–7)
Parity	—	1.8 ± 1.1 (0–6)
HPV16 Positive	114	76.0
HPV18 Positive	19	12.7
Other High‐Risk HPV	38	25.3
Pap Smear Normal	30	20.0
ASC‐US	28	18.6
LSIL	18	12.0
HSIL	8	5.3
ASC‐H	10	6.6
AGC	2	1.3
No Smear	54	36.0

### 3.2. Overall Colposcopy Performance

The contingency data of colposcopy findings versus LEEP histopathology are summarized in Table 2. Colposcopy demonstrated a sensitivity of 93.9% (95% CI: 87.3–97.4), specificity of 31.4% (95% CI: 20.2–45.0), and overall diagnostic accuracy of 72.7% (95% CI: 65.0–79.2). The PPV was 72.6% (95% CI: 64.2–79.7), and the NPV was 72.7% (95% CI: 51.9–86.9). The positive and negative likelihood ratios were 1.37 and 0.19, respectively. A total of 35 patients (23.3%) were false positives, undergoing LEEP despite not having HSIL on final histopathology.

**Table 2:** Overall colposcopy performance (total cohort, *n* = 150).

Colposcopy/LEEP	HSIL	Other	Total
HSIL	93	35	128
Other	6	16	22
Total	99	51	150
Performance Metric	Value	95% CI	—
Sensitivity	93.9%	87.3–97.4	—
Specificity	31.4%	20.2–45.0	—
Accuracy	72.7%	65.0–79.2	—
PPV	72.6%	64.2–79.7	—
NPV	72.7%	51.9–86.9	—
LR+	1.37	—	—
LR−	0.19	—	—

### 3.3. Subgroup Analysis – HPV16 Positive

In the HPV16‐positive subgroup, colposcopy sensitivity decreased to 80.8% (95% CI: 71.7–87.5), specificity remained low at 33.3% (95% CI: 21.9–47.1), and overall diagnostic accuracy was 64.7% (95% CI: 56.7–72.0). False‐positive LEEP procedures were slightly higher in this subgroup, suggesting that colposcopic interpretation may be more aggressive in HPV16‐positive patients. (Refer to Table 3 for HPV16 subgroup performance.)

**Table 3:** Colposcopy performance – HPV16 positive patients (*n* = 114).

Performance metric	Value	95% CI
Sensitivity	80.8%	71.7–87.5
Specificity	33.3%	21.9–47.1
Accuracy	64.7%	56.7–72.0
PPV	70.2%	61.2–78.1
NPV	47.2%	33.0–61.7
LR+	1.21	—
LR−	0.58	—

### 3.4. Cytology vs LEEP Correlation

Among patients with HSIL cytology, all 8 patients (100%) were confirmed as HSIL by LEEP. In the LSIL group, 18 patients were included, 7 of whom were upgraded to HSIL after LEEP. In the ASC‐US group, 28 patients were included, 4 of whom were upgraded to HSIL after LEEP. These results highlight that colposcopy alone may under‐ or overestimate lesion severity, emphasizing the importance of combining cytology, HPV status, and colposcopic findings in clinical decision‐making. (Refer to Table 4 for cytology vs LEEP correlation.)

**Table 4:** Correlation between Pap smear and LEEP results.

Cytology	LEEP HSIL	LEEP LSIL/other	Total
HSIL	8	0	8
LSIL	7	11	18
ASC‐US	4	24	28
Other/Normal	0	32	32

*Note:* This table shows the concordance between cytology and LEEP results, highlighting the rate of lesion upgrading or downgrading.

### 3.5. Clinical Implications

The observed high sensitivity but low specificity pattern indicates that colposcopy is effective as a rule‐out tool but carries a risk of unnecessary excisional procedures, particularly in tertiary referral centers. These findings underscore the importance of integrated risk assessment and careful clinical judgment to reduce overtreatment [5, 7, 10].

## 4. Discussion

The present study evaluated the diagnostic performance of colposcopy in a tertiary referral center and demonstrated a characteristic pattern of relatively high sensitivity accompanied by markedly low specificity. While the high sensitivity (93.9%) indicates that colposcopy is highly effective in identifying patients with HSIL, the low specificity (31.4%) raises important concerns regarding diagnostic precision and clinical decision‐making [5, 12, 13].

One of the most clinically relevant findings of this study is the substantial false‐positive rate (23.3%), indicating that nearly one in four patients underwent LEEP despite the absence of HSIL on final histopathological evaluation [4, 9]. This observation suggests potential overtreatment, particularly in tertiary referral settings where patient populations are enriched with high‐risk cases [6, 10, 14, 20]. In such settings, clinicians may adopt a more cautious or aggressive diagnostic approach to avoid missing high‐grade lesions, which may inadvertently lead to overestimation of disease severity [12, 17].

Previous studies have generally reported higher specificity rates for colposcopy, often exceeding 60%–90%, albeit with lower sensitivity [12–14, 21, 22]. In contrast, our findings demonstrate a reversed pattern, characterized by increased sensitivity at the expense of specificity. This discrepancy may be related to referral bias and differences in patient populations [5, 9, 18]. Tertiary centers typically manage more complex and higher‐risk cases, which may influence diagnostic behavior toward prioritizing sensitivity over specificity [6, 14].

A plausible explanation for the low specificity observed in this study relates to the inherent characteristics of colposcopic assessment in high‐risk populations. In tertiary referral centers, the pretest probability of high‐grade lesions is substantially elevated, which may lead clinicians to adopt a lower threshold for interpreting colposcopic findings as suspicious [6, 14]. This tendency, while improving sensitivity, inevitably increases false‐positive rates. In addition, the definition of a positive colposcopic result in this study may have further contributed to low specificity. Any colposcopic impression suspicious for HSIL was classified as positive, which may have lowered the diagnostic threshold and increased the number of false‐positive results. This approach reflects real‐world clinical practice in tertiary centers, where clinicians tend to prioritize sensitivity over specificity to avoid missing high‐grade lesions.

Additionally, colposcopy is a subjective, operator‐dependent procedure, and interobserver variability remains a well‐recognized limitation [5, 9, 18]. Subtle acetowhite changes, vascular patterns, and transformation zone characteristics may be overinterpreted, particularly in cases with concurrent high‐risk HPV positivity [9, 15]. In such contexts, even minor abnormalities may prompt classification as high‐grade lesions, contributing to reduced specificity.

Another contributing factor may be the overlap in colposcopic appearance between low‐grade and high‐grade lesions. Inflammation, metaplasia, and HPV‐related transient changes can mimic HSIL features, thereby increasing the likelihood of misclassification [12, 21]. This diagnostic ambiguity is further amplified in patients with HPV16 infection, where heightened clinical vigilance may bias interpretation toward more aggressive categorization [6, 15, 16].

Finally, the “see‐and‐treat” approach or a tendency toward defensive medicine in referral centers may also play a role [4, 17]. Clinicians may prefer to err on the side of overtreatment to avoid missing clinically significant lesions, particularly given the medico‐legal implications of delayed diagnosis. While this approach enhances detection rates, it comes at the cost of reduced specificity and increased unnecessary excisional procedures [4, 9, 10, 21].

Another important finding of this study is that HPV16 positivity did not improve the diagnostic performance of colposcopy. On the contrary, sensitivity decreased to 80.8% in HPV16‐positive patients, while specificity remained low. This unexpected result suggests that HPV16 status alone may not enhance the predictive value of colposcopic findings [6, 15, 16]. It is possible that the high baseline risk in HPV16‐positive patients leads to a lower threshold for interpreting lesions as high‐grade, thereby affecting diagnostic accuracy [9, 14].

From a clinical perspective, likelihood ratio analysis provides additional insight into the utility of colposcopy. The relatively low positive likelihood ratio (LR+ = 1.37) indicates that a positive colposcopic impression results in only a minimal increase in the probability of HSIL, suggesting limited usefulness for confirming disease. In contrast, the low negative likelihood ratio (LR− = 0.19) substantially reduces the probability of HSIL when colposcopy is negative, supporting its role as a rule‐out tool rather than a confirmatory diagnostic method. These findings highlight that colposcopy should not be used in isolation for treatment decisions but rather interpreted in conjunction with cytology, HPV status, and overall clinical risk assessment [5, 18].

The findings of this study have important clinical implications. Given the potential risks associated with LEEP, including bleeding, infection, cervical stenosis, and adverse obstetric outcomes [4, 10, 11, 17], minimizing unnecessary procedures is essential. The high false‐positive rate observed in this study highlights the need for more refined risk stratification strategies, potentially incorporating HPV genotyping, cytology, and adjunctive biomarkers [6, 7, 9, 14].

A notable limitation of this study is the absence of cervical cytology results in a considerable proportion of patients (36%). However, this finding reflects real‐world clinical practice in tertiary referral centers, where patients are frequently referred based on positive high‐risk HPV status or prior external assessments, often without complete cytological documentation. This study has several limitations. First, its retrospective design may introduce selection bias [1, 2]. In addition, since only patients undergoing LEEP were included, this represents a high‐risk population, which may have led to overestimation of sensitivity and underestimation of specificity. This limitation should be considered when interpreting the diagnostic performance of colposcopy. This limitation may influence the interpretation of cytology–histopathology correlations and should be considered when generalizing the findings. Second, the relatively small sample size may limit the generalizability of the findings and contribute to wider confidence intervals, particularly for specificity and NPV [12]. Third, the lack of patient‐level data precluded advanced statistical analyses such as multivariate regression [5, 9].

Despite these limitations, the study has notable strengths, including the use of a well‐defined patient cohort, consistency in clinical management within a single tertiary center, and histopathological evaluation by experienced pathologists [4, 6, 14].

## 5. Conclusion

Colposcopy demonstrates high sensitivity in detecting high‐grade cervical lesions; however, its low specificity may result in a considerable rate of overtreatment, particularly in tertiary referral settings [5, 8, 10]. These findings emphasize the importance of cautious interpretation of colposcopic findings and highlight the need for integrated, risk‐based diagnostic approaches to optimize patient management and reduce unnecessary excisional procedures [6–9, 18, 20].

## Funding

No funding was received for this manuscript.

## Conflicts of Interest

The author declares no conflicts of interest.

## Data Availability

The data that support the findings of this study are available upon request from the corresponding author. The data are not publicly available due to privacy or ethical restrictions.
